# The impact of participatory budgeting on health and wellbeing: a scoping review of evaluations

**DOI:** 10.1186/s12889-018-5735-8

**Published:** 2018-07-03

**Authors:** Mhairi Campbell, Oliver Escobar, Candida Fenton, Peter Craig

**Affiliations:** 1 MRC/CSO Social and Public Health Sciences Unit, University of Glasgow, 200 Renfield Street, Glasgow, G2 3QB UK; 2 What Works Scotland, Glasgow, UK; 3 University of Edinburgh, Edinburgh, UK

## Abstract

**Background:**

Participatory budgeting (PB), citizens deliberating among themselves and with officials to decide how to allocate funds for public goods, has been increasingly implemented across Europe and worldwide. While PB is recommended as good practice by the World Bank and the United Nations, with potential to improve health and wellbeing, it is unclear what evaluations have been conducted on the impact of PB on health and wellbeing.

**Methods:**

For this scoping review, we searched 21 databases with no restrictions on publication date or language. The search term ‘participatory budget’ was used as the relevant global label for the intervention of interest. Studies were included if they reported original analysis of health, social, political, or economic and budgetary outcomes of PB. We examined the study design, analysis, outcomes and location of included articles. Findings are reported narratively.

**Results:**

From 1458 identified references, 37 studies were included. The majority of evaluations (*n* = 24) were of PB in South America, seven were in Europe. Most evaluations were case studies (*n* = 23) conducting ethnography and surveys, focussing on political outcomes such as participation in PB or impacts on political activities. All of the quantitative observational studies analysing population level data, except one in Russia, were conducted in South America.

**Conclusion:**

Despite increasing interest in PB, evaluations applying robust methods to analyse health and wellbeing outcomes are scarce, particularly beyond Brazil. Therefore, implementation of PB schemes should be accompanied by rigorous qualitative and quantitative evaluation to identify impacts and the processes by which they are realised.

**Electronic supplementary material:**

The online version of this article (10.1186/s12889-018-5735-8) contains supplementary material, which is available to authorized users.

## Background

Participatory budgeting (PB) aims to democratically allocate public money for local services, enabling communities to decide how public funds are spent [1]. It entails a multi-stage process, which typically concludes with a vote, where citizens deliberate among themselves and with government officials to allocate funds for public goods [2]. Since it’s origination in 1989 in Porto Alegre, Brazil, PB has been implemented in many countries across North and South America, Europe and Asia, including many high income countries [3, 4]. International institutions including the World Bank, the Organisation for Economic Cooperation and Development (OECD), the United Nations and the UK Department for International Development recognise PB as good practice [3, 5, 6] and worldwide PB schemes distribute multimillion dollars/euros [7]. In Porto Alegre, PB has been reported to increase public spending in the poorest areas of the city, reduce administrative costs and improve citizen participation [8].

Literature on PB abounds, but to our knowledge, there has been no systematic assessment of the impact of PB on health and wellbeing across the world. PB may influence health and wellbeing via several pathways: increase in democratic participation; greater community cohesion; improved allocation of funding to public services prioritised by the community; and increased access to medical services via increased spending on healthcare and social determinants of health such as sanitation or housing. Table 1 outlines the possible stages of how PB can influence health and wellbeing, informed by key literature [3, 6, 9, 10].

**Table 1:** How PB can affect the health, social, democratic and economic outcomes of individuals

*The intervention, participatory budgeting, is expected to impact on the health, social and economic outcomes of individuals involved through the following stages, derived from UN-Habitat and World Bank reports (Cabannes 2004, Shah 2007) and (Boulding and Wampler 2010, page 126):*	
• ***Participation:*** *communities can decide how designated public money is spent.*	
• ***Collaboration:*** *being involved in the PB decision process enables citizens to exercise political rights, develop civic skills and build social cohesion.*	
• ***Prioritisation:*** *improvements in priority public services may improve the wellbeing of individuals in that community, either directly through impacts on their health (*e.g. *reduction in disease, better access to medical services) or* via *social determinants of health (*e.g. *housing, education).*	
• ***Allocation:*** *distribution of resources according to identified needs results in greater efficiency in the allocation of public funds, and greater accountability of budgetary procedures.*	

In order to establish the strength and reach of the existing evidence base for PB as a way of improving democratic participation, community cohesion, delivery of public services, and population health outcomes, we conducted a systematic scoping review of international literature to identify evaluations of PB schemes. Specifically, we examined what methods have been used to evaluate PB processes, what outcomes have been used to investigate the effects of PB, in which countries and at what geographic scale (e.g. neighbourhood, municipality, region). This scoping review is timely as there is growing interest from governments across Europe and beyond, in PB as a process for allocating public funds with the potential to address inequalities and increase community empowerment and cohesion [11, 12]. As PB is adapted and adopted in countries beyond Brazil, there can be less focus on social reform that was integral to the original process [13], with implications for benefits to health and wellbeing.

## Methods

The methodology for this review was guided by recent recommendations for conducting scoping reviews [14, 15], the protocol is available [16].

### Inclusion criteria

The review included studies of adults and children, as individuals or groups in communities involved in, or impacted by, PB. The intervention of interest was PB defined as communities deciding collectively how public funds are allocated. We did not include any form of individual budgetary decision--making (e.g. processes where individuals have personal management of welfare budgets, or employees have the opportunity to participate in defining their budget). Areas of interest included political, economic and budgetary, and health and social outcomes occurring at individual, population and systems levels. Political outcomes included outcomes at individual level such as participation, and systems level outcomes such as the democratic process at a local level. Economic and budgetary outcomes included outcomes at population level such as health or social service provision, housing, patterns of spending as the mechanism for impact on other outcomes, and provision of public goods (e.g. public parks, public safety). Health and social outcomes included outcomes at individual level such as health, wellbeing, self-efficacy, and empowerment, and at population level such as measurements of inequalities (e.g. poverty rates). We included any type of study design, quantitative, qualitative or mixed methods, with and without control groups or comparisons, which used primary analysis and reported on an evaluation of the PB system in relation to any of the outcomes listed above. This included peer reviewed published articles, books, reports and grey literature such as conference papers or working papers. There was no limitation on publication language or date; we did not find publications on PB prior to the 1980s when the process was established.

### Literature search

Twenty-one electronic databases were searched in October 2016, with an updated search conducted in May 2018. The databases included peer reviewed articles and grey literature, and aimed to cover a broad range of health, social, political and economic literature, see Additional file 1: Table S1 for full list. No filters or terms were used to capture evaluation studies as evaluation studies are not indexed as such in bibliographic databases. No language or date limits were applied to the literature searches. Further articles from the review authors’ collections were also included. The search term ‘participatory budget’ was used as this is the relevant global label used for the process that we wanted to examine; the term is used internationally to identify this specific process. The search results were first screened by title and abstract, with 10% independently screened by two reviewers. The full text was then screened, with duplicate screening of 10%, and disagreements resolved by discussion and in consultation with the third reviewer.

### Data extraction and collation

A data extraction template was developed in Microsoft Excel, tested and agreed by the review team. Duplicate data extraction was conducted on 20% of the included studies, see Additional file 1: Table S2 for data extraction template. As this was a scoping review, aiming to map the amount and type of evidence available on PB in relation to impacts on health and wellbeing, the literature was not formally appraised for methodological quality. The data were collated in summary tables and the results reported narratively.

## Results

**Fig. 1:**
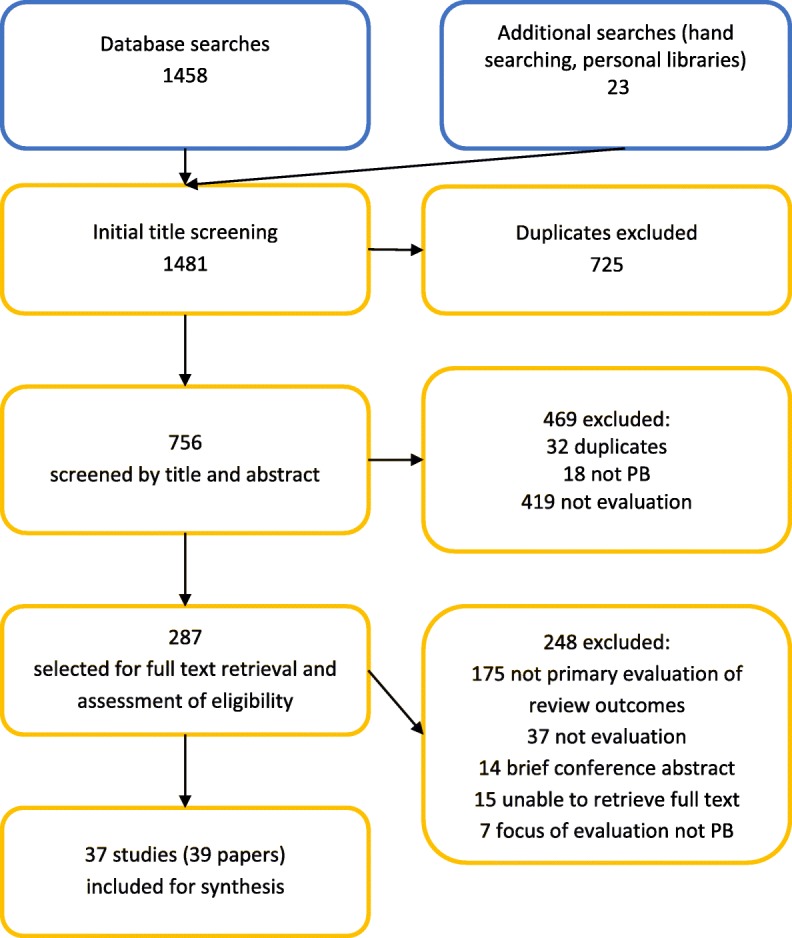
Literature screening flow diagram

The literature search identified 1458 citations. After de-duplication and screening, 39 articles reporting on 37 studies met the inclusion criteria, see Fig. 1 for a flow diagram of the screening process. A summary of characteristics table provides details of the included studies’ study design, country, data sources, methods of analysis, outcomes of interest, and source of funding (Table 2), and Additional file 1: Table S3 provides further details of the studies. Most of the studies that met the inclusion criteria were published in English; three of the included texts were in Portuguese and one in Spanish. Several evaluations had been published in English and the language of the country of origin.

**Table 2:** Included studies: study design, country, data sources, methods of analysis, and outcomes of interest

First author date	Country	Data source(s) Individual/aggregate data	Analysis	Outcome(s)	Source of funding for evaluation Investigator connection
Randomised controlled trials					
Beuermann 2014 [21]	Russia	Municipal data survey (*n* = 109) Household survey (*n* = 1645), political representatives (*n* = 109) Aggregate + Individual	Fixed effects regression comparing 2 PB treatment areas and control non-PB areas	Tax revenue. Allocation of PB funds. Participation in PB.	World Bank (IDB); Government of the Russian Federation. PB consultants for the RCT conducted evaluation
Quantitative observational studies					
Schneider 2002 [22]	Brazil	Municipal data (*n* = 497) Aggregate	Linear regression comparing PB and non-PB municipalities	Impact on participation in PB by spending of PB	None stated Academic investigator
Biderman 2007 [24]	Brazil	RAIS administrative records, census data Aggregate	Fixed effects regression comparing PB and non-PB municipalities	Tax revenue, infant mortality, education	Part funded by World Bank Academic investigators
Wampler 2007/2012 [30, 31]	Brazil	Survey (*n* = 833) Individual	Logistic regression	Impact on further political activities	National Science Foundation Academic investigator
World Bank 2008 [8]	Brazil	Municipal data (*n* = 150+) Survey (*n* = 1300) Aggregate + Individual	Difference in differences comparing PB and non-PB municipalities	Municipal budget PB expenditure, poverty, sanitation, infant mortality, education, participation of disadvantaged groups	Social Development Unit of the Latin America and Caribbean Region (LCSSO) and the Social Development Department (SDV) of the World Bank. NGO investigation
Boulding 2010 [9]	Brazil	Brazilian Institute of Geography and Economics, census Aggregate	Linear regression comparing PB and non-PB municipalities	Poverty, inequality (GINI), life expectancy, infant mortality, adult and child literacy. Allocation of PB funding	None stated. Academic evaluation
Garcia 2011 [29]	Spain	Center for Sociological Research Survey (*n* = 1004) Individual	Linear regression analysis comparing district council system	Participation of women, impact on further political activities	Women’s Institute and the National R & D & I Plan of the Government of Spain. Academic investigator
Borba 2012 [32]	Brazil	NUPESAL (Nucleo de Pesquisas Sobre a America Latina) Survey (*n* = 533) Individual	Logistic regression analysis comparing residents involved in PB and non-PB involved residents	Impact on further political activities, participation in PB	None stated Academic investigator
Jaramillo 2013 [23]	Peru	Municipal data (*n* = 219) from MSUNASS, PB-DNPP, MEF-SIAF, ONPE, JNE, RENAMU. Interviews (n =?) in 4 PB areas Aggregate + Individual	Linear regression analysis comparing 2007 data with 2001	Sanitation (water coverage and continuity)	Institutional Capacity Strengthening Fund (ICSF), managed by Inter-American Development Bank (IDB), Government of the People’s Republic of China. NGO investigation
Da Silva 2014 [33]	Brazil	Brazilian Institute of Geography and Statistics Aggregate	Cross tabulation descriptive analysis	PB investment per capita by deprived area. Impact on PB results by type of PB	Not stated Academic investigator
Goncalves 2014 [25]	Brazil	Municipal data (*n* = 3651) from Brazilian Institute of Geography and Economics, census Aggregate	Fixed effects regression comparing PB and non-PB municipalities	Municipal budget PB expenditure on education, housing, sanitation, cultural. Poverty, infant mortality	Not stated Academic investigator
Touchton 2014 [26]	Brazil	Municipal data (*n* = 253) from Brazilian Institute of Geography and Economics, census Aggregate	Random effects regression comparing PB and non-PB municipalities	Municipal budget PB expenditure on healthcare and sanitation. Infant mortality	Boise State University’s College of Social Science and Public Affairs Academic investigators
Jaramillo 2015 [27]	Peru	Municipal data Survey 2 wave (n = 100) Aggregate + Individual	Linear regression analysis comparing 2010 data with 2007	Number and quality of agricultural services	National Science Foundation and the Boren National Security Education Program Not for Profit research centre/academic investigators
Grillos 2017 [28]	Indonesia	Municipal data Aggregate	Linear regression analysis comparing city sub-districts	Allocation of PB funding	Harvard Kennedy School Indonesia Program Academic investigator
Single case studies					
Abers 1998 [34]	Brazil	Interviews (*n* = 90), survey (*n* = 622), participant observation in study area Individual	Qualitative	Mobilisation of neighbourhood residents. Impact on further political activities. Participation in PB by low income.	Not stated Academic investigator
Baiocchi 2001 [16]	Brazil	Survey (n = unclear, 10% plenary meeting attendees) Individual	Qualitative + descriptive quantitative	Participation in PB of disadvantaged groups (women, low income, low education).	National Science Foundation, Inter American Foundation, and University of Wisconsin Academic investigator
Baiocchi 2003 [47]	Brazil	Interviews (*n* = 65), survey (*n* = 74), participant observation at PB assemblies Individual	Qualitative	Use of PB assembly meetings for further community activities.	Not stated Academic investigator
Hernandez 2010 [41]	Brazil	Interviews (*n* = 30), participant observation at PB assemblies. Data from Data from Coordenadoria do Orçamento Participativo (COP) Individual	Qualitative + descriptive quantitative	Participation in PB of disadvantaged groups: Afro-Brazilians, senior citizens, children and adolescents, the GLBT community, women, indigenous groups, homeless, and people with disabilities.	Tinker Foundation, the Center for Latin American and Caribbean Studies and the Graduate School at Brown University Academic investigator
Walker 2013/2016 [44, 45]	Brazil	Interviews (n = 20), participant observation in study area Individual	Qualitative + descriptive quantitative	Political and social learning (negotiations for housing)	National Science Foundation and the Foundation for Urban and Regional studentship Academic investigators
Stewart 2014 [20]	USA	Municipal data (City of Chicago’s Aldermanic Menu reports), census data, PB evaluation reports Aggregate + Individual	Qualitative + descriptive quantitative	Participation in PB. Allocation of PB funds.	None stated Academic investigators
Célérier 2015 [36]	Brazil	Interviews (*n* = 18), survey (*n* = 46), participant observation	Qualitative	Participation in PB. Impact on political activities.	HEC Foundation and of the French Ministry of Foreign Affairs Academic investigators
Kendall 2015 [18]	Malawi	Survey, data collection (5 sites)	Qualitative + descriptive quantitative	Impact on democratic processes relating to the school, local communities and funding bodies.	TAG Philanthropic Foundation Academic investigators
McNulty 2015 [43]	Peru	Interviews (n = unclear), government data	Single case study	Participation in PB by women.	Franklin and Marshall College and The American Association of University Women Academic investigator
Hajdarowicz 2018 [63]	Columbia	Interviews (*n* = 19), participant observation	Qualitative	Participation in PB by women.	None stated Academic investigator
Multiple case studies					
Nylen 2003 [37]		Interviews (n = unclear), survey (*n* = 1280) Individual	Qualitative + descriptive quantitative	Participation in PB of disadvantaged groups (women, low education). Empowerment, impact on further political activities.	Academic investigator
Cabannes 2005 [49]	South America (multiple)	Survey (n = 4 PB schemes) ?	Qualitative + descriptive quantitative	Allocation of PB funds.	UNDP/Habitat NGO evaluation
Renno 2010 [51]	Brazil	Survey 3 wave (n = unclear)	Qualitative	Political learning. Impact on political activities.	None stated Academic investigator
SQW Consulting 2011 [19]	UK	Survey (n = unclear). Municipal data [check]	Qualitative + descriptive quantitative	Political and social learning	Department for Communities and Local Government Government evaluation
Wu 2011 [46]	China	Interviews (*n* = 15), Survey (*n* = 547)	Qualitative	Political and social learning	China Development Research Foundation (CDRF) Chinese central government, People’s Bank of China Academic investigators
Bassoli 2012 [48]	Italy	Survey 3 wave (2002/3 *n* = 12; 2005 *n* = 4; 2007/9 *n* = 9)	Qualitative	Democratic characteristics of PB schemes: inclusion, participation, the role of the opposition, and transparency.	None stated Academic investigator
Luchmann 2012 [42]	Brazil	Focus group PB delegates and councillors, survey (*n* = 47)	Qualitative	Political and social learning	CNPq (Conselho Nacional de Pesquisa / National Research Council) Academic investigator
Talpin 2012 [38]	Italy	Interviews (*n* = 12), participant observation at 54 PB meetings	Qualitative	Participation in PB. Political learning. Impact on further political activities.	None stated Academic investigator
Cabannes 2015 [39]	Multiple across South America, North America, Africa, Asia, Europe	Interviews (*n* = 12), Survey (*n* = 20) Aggregate + Individual	Qualitative + descriptive quantitative	Impact on political processes of municipality. Water supply, sanitation, public transport, roads, electricity supply	Aid UK, UK Government NGO (IIED) evaluation
del Prado 2015 [40]	Philippines	Interviews (*n* = unclear), focus groups (*n* = unclear). Municipal data (sources unclear) Individual	Qualitative + descriptive quantitative	Allocation of PB funds	Government think tank evaluation
Džinic 2016 [17]	Eastern Europe Multiple	Municipal data from municipality websites, press and official reports Aggregate	Qualitative + descriptive quantitative	Allocation of PB funds.	None stated Academic investigators
Montambeault 2016 [50]	Brazil	Survey (2009 *n* = 967, 2014 *n* = 473) Individual	Qualitative	Participation in PB.	Emerging Scholar grant from the Fonds de la Recherche du Québec—Société et Culture Academic investigator
Gregorčič 2016 [52]	Solvenia, Iceland	Interviews (n = 12, Solvenia), participant observation (Iceland)	Qualitative	Political and social learning	Not stated Academic investigator

### In which countries and policy sectors have PB processes been evaluated, and at what geographic scale?

The majority of the studies report on evaluations of PB in South America (*n* = 24), most in Brazil (*n* = 19), many (*n* = 7) focussed on Porto Alegre, where PB originated. The remaining South American studies were located in Peru (*n* = 3), Colombia (*n* = 1), and one multi-national study set in Brazil, Ecuador and Venezuela. There were seven evaluations of PB in Europe. One evaluation was conducted in the United States; three were set in Asia; and one study of PB was conducted in Africa. There was also one article collating case studies examining the impact of PB across twenty cities worldwide: South America (*n* = 12); Africa (*n* = 4); Europe (*n* = 2); Asia (*n* = 1); and the United States (*n* = 1).

The PB schemes operated and were evaluated at municipal level in 89% (*n* = 33) of the studies. The remaining four studies evaluated PB schemes at neighbourhood level [17–20]. The source of funding for the evaluation was reported in 59% of the studies; 38% government funding or non-government organisation, 21% reported an academic funding source. Academic researchers conducted 76% of the studies; the remaining 24% were non-government organisation evaluations.

### What methods have been used to evaluate PB processes?

The included studies were split broadly by study methodology and the level of data used. The first category, randomised controlled trials and quantitative observational studies, lists studies that used modelling to identify the effect of the outcomes or analysed large-scale population level data (Table 2). The second category collates case studies that conducted primary data collection in the form of surveys, interviews and participant observation; some of these studies included descriptive analysis of municipal data. Almost half of the case studies (*n* = 10) used mixed method study designs (qualitative interviews or surveys and descriptive quantitative analysis). One of the observational modelling studies included qualitative methods [8].

#### Randomised controlled trials and quantitative observational modelling studies

We found one randomised controlled trial (RCT). This three-arm trial, randomised by region and district, assessed the introduction of PB in rural settlements in Russia. The trial compared introduction of PB assisted by administrative training, to training plus additional technical assistance from two fulltime consultants, and included control districts that received neither training nor consultancy assistance [21].

There were thirteen quantitative observational studies. Nine studies analysed population level data using linear regression techniques [8, 9, 22–28]; three studies used regression models to analyse individual survey data [29–32], and one study used cross tabulation descriptive analysis to assess population level data [33].

#### Case studies

Twenty-three evaluations were case studies providing descriptive analyses of single (*n* = 10) or multiple (*n* = 13) instances of PB. The methods used for these case studies included participant observation (*n* = 9) [34–38], interviews (*n* = 15) [18, 34–46], surveys of PB participants and PB officials (*n* = 14) [18, 19, 34–37, 39, 42, 46–51], or descriptive analysis of municipal data (*n* = 6) [17, 19, 20, 40, 41, 43], fifteen studies applied various combinations of these methods.

### What outcomes have been used to investigate the effects of PB processes?

We define three broad categories of outcomes: political, economic and budgetary, and population outcomes (Table 3). The outcomes for each category are described below. While assessing the methodological quality of the studies was beyond the scope of this review, we include an indication of the results reported for interest (Additional file 1: Table S3).

**Table 3:** Cross-tabulation of study design by outcome category

Outcomes	Case studies single	Case studies multiple	Quantitative Observational Study (Population level data)	RCT
Population Social Impacts (poverty, health, education, housing, sanitation)	Kendall 2015 [18] Walker 2013/2016 [44, 45]		^a^Biderman 2007 [24] ^a^Boulding 2010 [9] ^a^Goncalves 2014 [25] ^a^Touchton 2014 [26] ^a^WorldBank 2008 [8]	
Economic and budgetary (funding of public services)	Stewart 2014 [20]	Cabannes 2005 [49] Cabannes 2015 [39] Del Prado 2015 [40] Dzinic 2016 [17]	Biderman 2007 [24] Boulding 2010 [9] Da Silva 2014 [33] Goncalves 2014 [25] Jaramillo 2013 [23] Jaramillo 2015 [27] Touchton 2014 [26] ^a^WorldBank 2008 [8] Grillos 2017 [28]	Beuermann 2014 [21]
Political (participation, democratic processes, political systems)	Abers 1998 [34] Baiocchi 2001 [47] Baiocchi 2003 [35] Célérier 2015 [36] Hernandez 2010 [41] ^a^Kendall 2015 [18] ^a^Stewart 2014 [20] ^a^Walker 2013/2016 [44, 45] Hajdarowicz 2018 [63]	Bassoli 2012 [48] ^a^Cabannes 2015 [39] Luchmann 2012 [42] McNulty 2015 [43] Montambeault 2016 [50] Nylen 2003 [37] Renno 2010 [51] SQW Consulting 2011 [19] Talpin 2012 [38] Wu 2011 [46] Gregorčič 2016 [52]	Borba 2012 [32] Garcia 2011 [29] Schneider 2002 [22] Wampler 2007/2012^b^ [30, 31] ^a^WorldBank 2008 [8]	

^a^ Study has outcomes in more than one outcome category

^b^ Wampler 2007/2012 analysis of survey data. Multiple dates indicates same data used in two articles

#### Political outcomes

Outcomes we labelled as ‘political’ were reported by two thirds (*n* = 26) of studies. These outcomes included: participation in PB by disadvantaged sections of the population; impacts on further involvement in political or civic activities; and learning about political processes as a result of engagement in PB. The majority (*n* = 20) of political outcomes were evaluated by descriptive case studies. Thirteen studies examined participation in PB of disadvantaged groups including women, people with low income, low education, disability, or of an ethnic minority, with overall mixed results reported. Several observational studies reported some increase in participation [8, 21, 22, 29, 32]. The case studies reported more equivocal findings, levels of participation by disadvantaged groups was increased [37], low [20, 47] or mixed [41]; barriers remained [43] and active participation (i.e. taking part in discussions) was found only to increase with increased years of involvement in PB [38, 50].

The relationship between PB and further involvement in political or civic activities was explored in six studies [30, 31, 34, 35, 37, 42, 51], sometimes reporting that the individuals engaging in PB were already involved in other civil society organisations. Changes in how people acted in political arenas as a result of PB were examined by nine studies [18, 30, 32, 36, 39, 42, 44, 46, 48], tending to report positive impacts. Learning about political processes as a result of engagement in PB was examined by four case studies [19, 37, 42, 52], in general reporting some increased learning of political processes.

#### Economic and budgetary outcomes

Twelve studies reported on economic or budgetary outcomes such as local tax revenue or local government spending on public services such as healthcare, sanitation and housing. Two studies analysed impacts on local tax revenue from municipal records [21, 24], using fixed effect regression analysis to examine whether there was a change in local tax revenue according to the implementation of PB, reporting positive results.

Thirteen studies examined the allocation of PB funds. Three studies set in Brazil assessed spending on healthcare and sanitation, comparing municipalities that implemented PB with comparable areas that did not, reporting positive results [9, 25, 26]. All three studies applied linear regression analysis to large datasets: for all Brazilian municipalities with available data [25]; and for municipalities in Brazil with a population of over 100,000 inhabitants, 220 municipalities from 1991 to 2000 [9] and 253 municipalities between 1989 and 2008 [26]. Analysis of PB in Indonesia found areas with more low income households were less likely to benefit from the PB process [28]. Linear regression was also used to examine allocation of PB funding in relation to citizens’ priorities in Russian settlements [21]. The impact of PB on the number and quality of agricultural services provided in 100 randomly selected Peruvian municipalities was assessed using linear regression analysis of data from the municipalities and central government [27]. The impact of PB on the provision of water services in 219 districts in Peru was analysed using linear regression, reporting no improvement to the services [23]. Budgetary expenditure on public services including water and sanitation in Porto Alegre in Brazil was analysed using difference in difference regression analysis, reporting positive results [8]. Municipal budgetary and census data from the Brazilian Institute of Geography and Statistics was used to conduct a descriptive analyse of the allocation of PB funds according to level of deprivation, finding mixed results [33]. Four further studies examined which public services received funding from PB, which included provision of recreational public spaces [17, 20, 49], and micro credit centres [40].

#### Health and social outcomes

Health or social outcomes, such as infant mortality, poverty rates, education, and access to sanitation, were evaluated by eight studies, most studies examining multiple outcomes. The impact of PB on infant mortality was examined by five quantitative observational studies located in Brazil [8, 9, 24–26]. These studies report mixed results. The two more recent studies reported that PB reduced infant mortality [25, 26]. Poverty rates in municipalities implementing PB were assessed by two studies in Brazil, both of which reported reductions in poverty [8, 9]. Education, measured as either child or adult literacy or years of school attendance, was investigated by two quantitative observational studies and one case study, with mixed results [9, 18, 24]. Access to sanitation, piped water supply and sewerage, was measured by one observational study and one case study, reporting positive results [8, 39].

## Discussion

This scoping review provides a systematic analysis of studies evaluating health and wellbeing impacts of PB. We identified 37 evaluation studies focussing on issues relating to health and wellbeing or delivery of public services, a relatively small number in comparison with the estimated 2000 to 2700 PB schemes implemented worldwide [11, 53]. While a few evaluations used population level datasets to examine health outcomes in South America, overall there was limited scope in the methods used to assess PB, and the vast majority of studies focused on political outcomes.

The majority of studies found were single or multiple case studies, describing the scenario of individual PB schemes. There were fewer observational quantitative modelling studies using large population datasets, and only one study that combined this with qualitative analysis. This dearth of mixed methods approaches is somewhat puzzling. PB interventions are intended to realise a complex range of democratic and social goods through both the processes and outcomes of public participation in budgetary decision-making. Mixed methods approaches, with their capacity to combine exploratory and explanatory research designs [54, 55], are uniquely suited to conduct multidimensional evaluations of both processes and outcomes [56]. While further detail of mixed methods may be included in book length descriptions of PB processes (e.g. [57–59]), our review suggests that there is considerable scope for methodological development and innovation using mixed methods approaches to evaluate PB.

The majority of studies identified in this review were evaluations of PB in South America. All of the quantitative observational studies analysing population level data were conducted in South America, with the exception of the randomised controlled trial conducted in Russia. Evaluation of PB in Brazil is possible due to the availability of population data aggregated at municipal level. Such data is not routinely available in many countries [60]. Robust evaluation of PB in other countries may require developing databases at local government level. The concentration of PB evaluation in Brazil has strong implications for policy makers in countries beyond South America interested in implementing PB. As PB has been developed in other continents, and in high income countries, the processes, budgets, and scale of the initiatives have become more varied [13, 61]. Also, the rationale of social justice underpinning PB when it began in Porto Alegre has often become marginalised in PB outside Brazil [13]. While the results of PB in Brazil may be generalizable to some other countries with similar levels of existing public services, in general, these differences suggest that the results of evaluations on health and wellbeing outcomes found in PB in Brazil may not translate to PB in other contexts. The main focus of PB evaluations was on political outcomes, we found less evaluation of outcomes relating to delivery of public services or assessing health outcomes or wellbeing outcomes related to poverty. In part, this may relate to who is conducting the analysis; as far as we could determine, the majority of studies were conducted within the fields of political science and public administration. Despite worldwide implementation of PB, this review finds that the implications for health and wellbeing have not been the focus of attention in public health literature. In our review, the exception is provided by Boulding and Wampler [9], who discuss the possible impacts on wellbeing, finding limited research in this area. More recently, studies have accessed population level datasets to investigate health impacts, focussing on outcomes such as infant mortality, education and poverty rates (e.g. [25, 26]). However, the limited evaluations of PB health and wellbeing outcomes may also relate to the ad hoc quality of many PB processes. Only Brazil has institutionalised PB to the point where comparative and longitudinal evaluations become viable, which accentuates the challenge of assessing health and wellbeing outcomes. Nevertheless, recent policy developments in other countries (e.g. Harkins et al. [12, 13, 62]) are seeking to embed PB in local government for the long term. This offers the opportunity for a PB evaluation agenda that transcends the geographic and thematic foci prevalent in the field.

### Strengths and limitations of this review

We used systematic, transparent methods with predetermined inclusion criteria. It is possible that some articles may not have been identified in the literature search, however, we are confident that the search of twenty-one databases provided us with a broad sweep of international peer-reviewed articles and grey literature on PB. As this was a scoping review, we did not make a formal assessment of the risk of bias in the included studies. Our aim was to map out what evaluations relating to health and wellbeing have been conducted. This section outlines key learning points and implications, in particular regarding the methodological, geographic, and thematic foci that dominate the field of PB evaluations.

## Conclusion

The findings of this review lead us to recommend further evaluation of the impact of PB on health and wellbeing be conducted in a range of national contexts. The lack of substantial evaluations outside Brazil, and the variations of PB being implemented worldwide, support the recommendation that when PB is being adopted, adapted and initiated, this should be accompanied by rigorous evaluation of the process and expected outcomes, using appropriate comparators. Governments involved in supporting and developing PB processes are uniquely placed to ensure that evaluation is not an afterthought, but an embedded component of robust PB policy over the long term. PB has spread globally, partly on the basis of claims regarding its potential to empower communities and improve people’s lives. Our review indicates that the PB field needs a stronger evidence base in order to substantiate and refine those claims in a variety of contexts.

## Additional file

Additional file 1: **Tables S1a** and **S1b** Databases searched. **Table S2** Data extraction template. **Table S3** Detailed characteristics of studies. (DOCX 30 kb)

## Abbreviations

PB: Participatory budgeting
UK: United Kingdom
