# Supplementary material for: The impact of participatory budgeting on health and wellbeing: a scoping review of evaluations
Source: BMC Public Health. 2018 Jul 3;18:822. doi: 10.1186/s12889-018-5735-8 (PMC6029380; doi:10.1186/s12889-018-5735-8)
Supplement: Supplementary file 1 — Tables S1a and S1b Databases searched. Table S2 Data extraction template. Table S3 Detailed characteristics of studies. (DOCX 30 kb) [file 12889_2018_5735_MOESM1_ESM.docx]

**The impact of Participatory Budgeting on health and wellbeing: a scoping review of evaluations**

**Supplementary data**

- **Table S1a and S1b: Databases searched**
- **Table S2: Data extraction template**
- **Table S3: Detailed characteristics of studies**

**Table S1a: Databases searched**

Participatory budgeting search diary 19.10.16. Search phrase used across all databases "Participatory budget*"

| **Database** | **Date searched** |
| --- | --- |
| America: History & Life | 10.10.16 |
| ASSIA | 10.10.16 |
| Child Development & Adolescent Studies | 10.10.16 |
| CINAHL | 10.10.16 |
| Embase | 10.10.16 |
| GreenFILE | 10.10.16 |
| Health Source: Nursing/Academic Edition | 10.10.16 |
| IBSS | 10.10.16 |
| Medline | 10.10.16 |
| Open Grey | 12.10.16 |
| Planex | 18.101.6 |
| PsycINFO | 10.10.16 |
| Scopus | 10.10.16 |
| Social Services Abstracts | 12.10.16 |
| SocINDEX with Full Text | 10.10.16 |
| Sociological Abstracts | 19.10.16 |
| Web of science | 10.10.16 |
| WHOLIS | 12.10.16 |
| Worldwide Political Science Abstracts | 12.10.16 |
|  |  |
| Econlit | 19.10.16 |
| Econpapers | 19.10.16 |
|  |  |

**Table S1b: Updated searches**

| **Database** | **Date searched** |
| --- | --- |
| America: History & Life | 21.05.18 |
| IBSS | 21.05.18 |
| Open Grey | 21.05.18 |
| Scopus | 21.05.18 |
| SocINDEX with Full Text | 21.05.18 |
| Sociological Abstracts | 21.05.18 |
| Web of science | 21.05.18 |
| Worldwide Political Science Abstracts | 21.05.18 |
| Google Scholar | 21.05.18 |

**Table S2: Participatory Budgeting scoping review: data extraction template**

| **Author Year Title** | *First author name, year published, article title* |
| --- | --- |
| **Data extracted by** | *Reviewer initials* |
| **Aim of study** | *Note aim of study (focus of study can help interpret/put study in context for our review)* |
| **Study design** | *Note details of study design: Quantitative (including economic evaluations), Qualitative, Mixed* |
| **Evaluation methodology** | *Note details of methodology, e.g.*   - *experimental (e.g. RCT)* - *quasi-experimental (e.g. controlled study, pre-test post-test control group study)* - *observational analytic (e.g. cohort study)* - *observational descriptive (e.g. case study)* - *expert opinion (e.g. expert consensus, bench research)* |
| **Intervention** | *Note details of PB scheme being evaluated (record ‘not stated’ if no information provided)*   - *Level (e.g. local council, local government, regional etc.)* - *Range of budgets included in PB scheme(amount, policy etc)* - *Who participated* |
| **Population** | *Note details of population group(s)*  *including any information on demographic breakdown, such as by sex, age, ethnic group, socioeconomic group* |
| **Setting** | *Note details of country, region, city; level of government; policy sector implementing the PB scheme.* |
| **Outcomes** | *Note details of any outcomes relating directly or indirectly to health, social, political, or economic impacts of participatory budgeting. This may include, but not limited to, individual outcomes, social determinants of health, and systems outcomes such as any recorded impact on changes to public service provision or further political processes.* |
| **Results** | *Note summary of results here* |
| **Funder / Investigator** | *Note details of funding source (record ‘not stated’ if no information provided)*  *Details of investigator: academic, NGO, government* |
| **Comments** |  |

**Table S3: Table of detailed characteristics of included studies: study design, country, data sources, methods of analysis, outcomes of interest, brief results, and source of funding**

| **Author date** | **Country** | **Data source(s)**  **Individual/aggregate data** | **Analysis** | **Outcome(s)** | **Author reported results** | **Source of funding for evaluation**  **Investigator connection** |
| --- | --- | --- | --- | --- | --- | --- |
| **Randomised controlled trials** | | | | | | |
| (Beuermann and Amelina 2014) | Russia | Municipal data survey (n=109)  Household survey (n=1645), political representatives (n=109)  Aggregate + Individual | Fixed effects regression comparing 2 PB treatment areas and control non-PB areas | Tax revenue. Allocation of PB funds. Participation in PB. | Positive for full treatment arm in pre-established ‘settlement’ areas | World Bank (IDB); Government of the Russian Federation.  PB consultants for the RCT conducted evaluation |
| **Quantitative observational studies** | | | | | | |
| (Schneider and Goldfrank 2002) | Brazil | Municipal data (n=497)  Aggregate | Linear regression  comparing PB and non-PB municipalities | Impact on participation in PB by spending of PB | Increase in budgets in areas with higher PB participation. Higher spending in more deprived areas. | None stated  Academic investigator |
| (Biderman and Silva 2007) | Brazil | RAIS administrative records, census data  Aggregate | Fixed effects regression comparing PB and non-PB municipalities | Tax revenue, infant mortality, education | Positive impact on tax revenues, years of schooling. PB municipalities spend more on health to achieve same change in infant mortality as non-PB municipality. | Part funded by World Bank  Academic investigators |
| (Wampler 2007, Wampler 2012) | Brazil | Survey (n=833)  Individual | Logistic regression | Impact on further political activities | Positive changes to political activities, participatory practices incorporated in arenas outside PB. Residents in communities receiving public services via PB likely to be involved with CSOs. Longer timescale of PB positively linked to residents’ belief in their ability to influence policy decisions. | National Science Foundation  Academic investigator |
| (World Bank 2008) | Brazil | Municipal data (n=150+)  Survey (n=1300)  Aggregate + Individual | Difference in differences  comparing PB and non-PB municipalities | Municipal budget PB expenditure, poverty, sanitation, infant mortality, education, participation of disadvantaged groups | Good representation of women, low-income groups, ethnic minorities and people with disabilities. Under-represented: extremely poor, youth, middle- and high income groups. Improved public understanding of 'pro-poor spending', engaging public in expressing priorities for capital spending but no improvement in budget literacy. Poverty rates reduced in PB. Access to piped water and sewerage increased. PB had to be implemented for over 10 years for positive impacts (before 1996). Other non-PB state capitals (weighted) reduced infant mortality at faster rate than PA - however, PA infant mortality at lower rate at baseline. | Social Development Unit of the Latin America and  Caribbean Region (LCSSO) and the Social Development Department (SDV) of the World Bank.  NGO investigation |
| (Boulding and Wampler 2010) | Brazil | Brazilian Institute of Geography and Economics, census  Aggregate | Linear regression  comparing PB and non-PB municipalities | Poverty, inequality (GINI), life expectancy, infant mortality, adult and child literacy. Allocation of PB funding | Small positive effect on extreme poverty, increase in healthcare and sanitation spending. No effect for all other outcomes | None stated. Academic evaluation |
| (Garcia, Mora et al. 2011) | Spain | Center for Sociological Research  Survey (n= 1004)  Individual | Linear regression analysis  comparing district council system | Participation of women, impact on further political activities | PB reduces the ‘double call effect’; reduction in proportion of men and those previously participating in political/civic society compared to traditional models such as District Councils. | Women's Institute and the National R & D & I Plan of the Government of Spain.  Academic investigator |
| (Borba and Ribeiro 2012) | Brazil | NUPESAL (Nucleo de Pesquisas Sobre a America Latina)  Survey (n=533)  Individual | Logistic regression analysis comparing residents involved in PB and non-PB involved residents | Impact on further political activities, participation in PB | Participation in PB over several PB years found to impact positively on interpersonal trust, electoral participation and participation in associations; taking part in PB neutralised the demobilising effect of low income and education. PB resulted in trust in municipal institutions and interest in politics. Participation in PB over time had similar or greater impact on development of civic attitudes as level of education. | None stated  Academic investigator |
| (Jaramillo and Alcázar 2013) | Peru | Municipal data (n=219) from MSUNASS, PB-DNPP, MEF-SIAF, ONPE, JNE, RENAMU.  Interviews (n=unclear) in 4 PB areas  Aggregate + Individual | Linear regression analysis comparing 2007 data with 2001 | Sanitation (water coverage and continuity) | No statistically significant association between PB and water coverage or service continuity. PB process found to have limitations in ability to channel resources to basic services, including water and sanitation. | Institutional Capacity Strengthening Fund (ICSF), managed by Inter-American  Development Bank (IDB), Government of the People’s Republic of China.  NGO investigation |
| (da Silva 2014) Da Silva 2014 | Brazil | Brazilian Institute of Geography and Statistics  Aggregate | Cross tabulation descriptive analysis | PB investment per capita by deprived area. Impact on PB results by type of PB | In 2002-2005, PB investment favoured disadvantaged regions that lacked public services and goods, due to the redistributive weighting system of the PB process. In the period 2005-2008, redistributive effects decreased, with disadvantaged regions receiving less investment that in the first period. | Not stated  Academic investigator |
| (Goncalves 2014) | Brazil | Municipal data (n=3651) from Brazilian Institute of Geography and Economics, census  Aggregate | Fixed effects regression comparing PB and non-PB municipalities | Municipal budget PB expenditure on education, housing, sanitation, cultural. Poverty, infant mortality | Increase in expenditure on health and sanitation. Reduction in infant mortality associated with municipal level increase in share of expenditure allocated to health and sanitation. | Not stated  Academic investigator |
| (Touchton and Wampler 2014) | Brazil | Municipal data (n=253) from Brazilian Institute of Geography and Economics, census  Aggregate | Random effects regression comparing PB and non-PB municipalities | Municipal budget PB expenditure on healthcare and sanitation. Infant mortality | Decrease in infant mortality per 1000 live births, greater decrease in municipalities with PB for 8+ years. | Boise State University’s College of Social Science and Public Affairs  Academic investigators |
| (Jaramillo and Wright 2015) | Peru | Municipal data  Survey 2 wave (n=100)  Aggregate + Individual | Linear regression analysis comparing 2010 data with 2007 | Number and quality of agricultural services | Municipalities having PB was found to have a positive impact on the number of agricultural services provided. However, the effectiveness of the services was judged to be less effective than processes in place in 2001 prior to mandatory PB. | National Science Foundation and the Boren National Security Education Program  Not for Profit research centre/academic investigators |
| (Grillos 2017) | Indonesia | Municipal data  Aggregate | Linear regression analysis comparing city districts | Poverty | Households in poverty less likely to benefit from the PB process. This was due to greater involvement by more affluent areas at the proposal stage of the PB process. | Harvard Kennedy School Indonesia Program  Academic investigator |
| **Single case studies** | | | | | | |
| (Abers 1998) | Brazil | Interviews (n=90), survey (n=622), participant observation in study area  Individual | Qualitative | Mobilisation of neighbourhood residents. Impact on further political activities. Participation in PB by low income. | PB resulted in large increase in neighbourhood activism - in poorest neighbourhoods of city. New neighbourhood organisations created, mobilisation of residents to attend PB, increase in residents’ involvement in civic social organisations and alliances within and between neighbourhoods. | Not stated  Academic investigator |
| (Baiocchi 2001) | Brazil | Survey (n=unclear, 10% plenary meeting attendees)  Individual | Qualitative + descriptive quantitative | Participation in PB of disadvantaged groups (women, low income, low education). | Active participation less likely by women, low income or education, however this negated by time involved in PB. | National Science Foundation, Inter American Foundation, and University of Wisconsin  Academic investigator |
| (Baiocchi 2003) | Brazil | Interviews (n=65), survey (n=74), participant observation at PB assemblies  Individual | Qualitative | Use of PB assembly meetings for further community activities. | PB meetings used as arena for personal and broader community issues | Not stated  Academic investigator |
| (Hernandez-Medina 2010) | Brazil | Interviews (n=30), participant observation at PB assemblies. Data from Data from Coordenadoria do Orçamento Participativo (COP)  Individual | Qualitative + descriptive quantitative | Participation in PB of disadvantaged groups: Afro-Brazilians, senior citizens, children and adolescents, the GLBT community, women, indigenous groups, homeless, and people with disabilities. | Increase of disadvantaged groups as delegate members. Increased inclusion of Afro-Brazilians, senior citizens, youth, women, indigenous groups, homeless people. Decrease of GLBT community and people with disabilities proportional decrease. | Tinker Foundation, the Center for Latin American and Caribbean Studies and the Graduate School at Brown University  Academic investigator |
| (Walker 2013, Walker 2016) | Brazil | Interviews (n=20), participant observation in study area  Individual | Qualitative + descriptive quantitative | Political and social learning (negotiations for housing) | PB enabled housing planners and residents to better co-manage conflicts through joint decision-making and negotiating to meet diverse needs of diverse groups of residents. PB enabled women to develop skills as community leaders. However, conflict about plans to erect new housing can cause delays for substantial amounts of time (example 5 years). | National Science Foundation and the Foundation for Urban and Regional studentship  Academic investigators |
| (Stewart, Miller et al. 2014) | USA | Municipal data (City of Chicago’s Aldermanic Menu reports), census data, PB evaluation reports  ([www.pb49.org](http://www.pb49.org))  Aggregate + Individual | Qualitative + descriptive quantitative | Participation in PB. Allocation of PB funds. | While efforts were made for PB to be inclusive (various locations of meetings, use of Spanish at meetings) tiny minority of ward population participated; vast majority of those who participated were 'white', in contract to the ethnic diversity of the ward. | None stated  Academic investigators |
| (Célérier and Cuenca Botey 2015) | Brazil | Interviews (n=18), survey (n=46), participant observation | Qualitative | Participation in PB. Impact on political activities. | Being a PB councillor gave skills and dispositions that increased their social status, giving them opportunity to influence social change as part of a more dominant group, but also led to some alienation from their original social groups. | HEC Foundation and of the French Ministry of Foreign Affairs  Academic investigators |
| (Kendall, Kaunda et al. 2015) | Malawi | Survey, data collection (5 sites) | Qualitative + descriptive quantitative | Impact on democratic processes relating to the school, local communities and funding bodies. | PB improved local accountability and school-community relations. | TAG Philanthropic Foundation  Academic investigators |
| (McNulty 2015) | Peru | Interviews (n=unclear), government data | Single case study | Participation in PB by women. | Women prevented from attending PB meetings by cultural and economic factors (high poverty, cultural norms about women’s duties). Poverty worse for women in rural areas, lack of education in poorer areas impacts on understanding PB processes. | Franklin and Marshall College and The American Association of University Women  Academic investigator |
| (Hajdarowicz 2018) | Colombia | Interviews (n=19), participant observation | Qualitative | Participation in PB by women. | Women reported increased knowledge of political processes, increased involvement in community issues, and personal capacity to tackle situations such as domestic violence. | None stated  Academic investigator |
| **Multiple case studies** | | | | | | |
| (Nylen 2003) |  | Interviews (n=unclear), survey (n=1280)  Individual | Qualitative + descriptive quantitative | Participation in PB of disadvantaged groups (women, low education). Empowerment, impact on further political activities. | More women and people with low level of educational qualifications active in PB than other political institutions such as local councils. PB not increasing engagement in other SCO activities; tendency for people involved in PB were already active in CSOs. PB activities enhanced democratic representation through open: election of PB delegates; allocation of goods and services; and accountability and operations of the relevant municipal departments. | Academic investigator |
| (Cabannes 2005) | South America (multiple) | Survey (n=4 PB schemes) | Qualitative + descriptive quantitative | Allocation of PB funds. | PB found to result in prioritisation of provision of basic services and strong mechanism for delivery of such services at local level. A third of all projects and half of total spending were on basic service provision. Variation in proportion of basic services projects, tending to be more numerous in poor and recent PB cities. | UNDP/Habitat  NGO evaluation |
| (Renno and Ames 2010) | Brazil | Survey 3 wave (n=unclear) | Qualitative | Political learning. Impact on political activities. | PB activists not necessarily better informed about politics than non-activists. Gender gap in political information still exists in PB activists. PB activists' networks larger. PB activists tend to be more engaged in other forms of social and political organisations. Activists tend to have specific characteristics regarding partisanship and ideology - different from rest of population. | None stated  Academic investigator |
| (SQW Consulting 2011) | UK | Survey (n=unclear). Municipal data | Qualitative + descriptive quantitative | Political and social learning | PB had potential to: improved individuals' and organisations' confidence in dealing with neighbourhood issues and negotiating with public sector organisations. Encourage people to create and develop community organisations, and become more involved in existing community organisations. Raise councillor profiles, improve communication with councillors, improve councillor understanding of ward issues. Increase confidence in local service providers. | Department for Communities and Local Government  Government evaluation |
| (Wu and Wang 2011) | China | Interviews (n=15), Survey (n=547) | Qualitative | Political and social learning | Satisfaction with: communication between the government and the public, project selection procedure and improvement on financial transparency of the government. Less satisfaction with process for electing resident representatives and the method of deliberative discussion in the project selection process. Improvements in: performance of government officials, relationship between the government and the public, allocation of funds for public identified priority projects, and helping the public to better understand policies. | China Development Research Foundation (CDRF) Chinese central government, People's Bank of China  Academic investigators |
| (Bassoli 2012) | Italy | Survey 3 wave (2002/3 n=12; 2005 n=4; 2007/9 n=9) | Qualitative | Democratic characteristics of PB schemes: inclusion, participation, the role of the opposition, and transparency. | PB schemes tended to have broad inclusion criteria, e.g. under-age citizens and non-formal citizens. Self-selection was a problem, factional participation according to political divisions. Citizens tended to be aware of their opportunities to participate in PB. Tended to be agreement of priorities between PB administrators resulting in issues voted for included in the final budget. Opposition parties tended not to be involved in PB, with the projects developed by the centre-left majority political parties. | None stated  Academic investigator |
| (Luchmann 2012) | Brazil | Focus group PB delegates and councillors, survey (n=47) | Qualitative | Political and social learning | Positive impact on delegates and councillors’ knowledge/information gains and capacity to deliberate on priorities across neighbourhoods and the region. Increased interest in politics and political self-efficacy. Increase in participation in processes beyond PB. Low levels of interpersonal trust. PB elicited changes in existing civic associations and emergence of new ones that reinvigorate local politics. However, municipal authorities do not set out to use PB for developing civic culture but to improve local governance. | CNPq (Conselho Nacional de Pesquisa / National Research Council  Academic investigator |
| (Talpin 2012) | Italy | Interviews (n=12), participant observation at 54 PB meetings | Qualitative | Participation in PB. Political learning. Impact on further political activities. | A fifth of participants never spoke at observed PB working groups, therefore had limited involvement. Majority of participants did not learn how to be significant 'good citizens' i.e. being able to use language of the PB institution and be seen to reflect general interest not just personal gain. High turnover rate of participants, many of whom become cynical of participatory democracy as well as politics in general; their PB experience moving them from being apolitical to anti-political. | None stated  Academic investigator |
| (Cabannes 2015) | Multiple across South America, North America, Africa, Asia, Europe | Interviews (n=12), Survey (n=20)  Aggregate + Individual | Qualitative + descriptive quantitative | Impact on political processes of municipality. Water supply, sanitation, public transport, roads, electricity supply | While PB tended to improve governance, not found to alter 'existing power relations between government and citizens'. PB tended to help empower citizens, instigate new community organisations, and through joint decision making bodies 'strengthen societal governance'. | Aid UK, UK Government  NGO (IIED) evaluation |
| del Prado, Rosellon et al. (2015) | Philippines | Interviews (n=unclear), focus groups (n=unclear). Municipal data (sources unclear)  Individual | Qualitative + descriptive quantitative | Allocation of PB funds | Around half of the PB budget in each municipality was assigned to CSO proposed livelihood projects such as trading centres, micro credit centres. Increase in number of CSOs participating in PB. | Government think tank evaluation |
| (Džinic, Murray et al. 2016) | Eastern Europe  Multiple | Municipal data from municipality websites, press and official reports  Aggregate | Qualitative + descriptive quantitative | Allocation of PB funds. | Town budgets assigned for PB, allocated to road construction, sports fields, sewage tank, street lighting, modernisation of recreation places, creation of community gardens, playground, revitalising public space, public bike rent. Very small proportion of public budgets allocated to PB. | None stated  Academic investigators |
| (Montambeault 2016) | Brazil | Survey (2009 n=967, 2014 n=473)  Individual | Qualitative | Participation in PB. | People new to PB less likely to actively participate. High attrition rate and stable participation figures suggest flow of non-active participants, not becoming citizen agents, rather citizen users (get what want and stop being involved). New participants less likely to speak at discussion meetings than those involved for 8+ years, large proportion who never took part in discussions, this was linked to low educational attainment. | Emerging Scholar grant from the Fonds de la Recherche du Québec—Société et Culture  Academic investigator |
| (Gregorcic 2016) | Solvenia, Iceland | Interviews (n=12, Solvenia), participant observation (Iceland) | Qualitative | Political and social learning | Interviewees reported increased learning about public funding and politics, and how to discuss and address the needs of their community | Not stated  Academic investigator |
